# *Escherichia coli* as a Presentation of Fulminant Early-Onset Neonatal Sepsis

**DOI:** 10.3390/jcm15052000

**Published:** 2026-03-05

**Authors:** Norbert Dera, Kacper Dera, Natalia Żeber-Lubecka, Michał Ciebiera

**Affiliations:** 1 Department of Obstetrics, Perinatology and Neonatology, Center of Postgraduate Medical Education, 01-809 Warsaw, Poland; 2 Department of Neonatology and Neonatal Pathology, Warsaw Institute of Women’s Health, 00-189 Warsaw, Poland; 3 Department of Pediatric Cardiology and General Pediatrics, Medical University of Warsaw, 02-091 Warsaw, Poland; 4 Department of Gastroenterology, Hepatology and Clinical Oncology, Center of Postgraduate Medical Education, 02-781 Warsaw, Poland; 5 Department of Genetics, Maria Sklodowska-Curie National Research Institute of Oncology, 02-781 Warsaw, Poland; 6 Second Department of Obstetrics and Gynecology, Centre of Postgraduate Medical Education, 00-189 Warsaw, Poland; 7 Department of Gynecology, Warsaw Institute of Women’s Health, 00-189 Warsaw, Poland

**Keywords:** sepsis, neonate, PPROM, *Escherichia coli*

## Abstract

**Background:** Neonatal sepsis is a bloodstream infection and constitutes a major cause of morbidity and mortality among newborns. The diagnosis is primarily based on a positive blood culture result. An additional diagnostic and therapeutic challenge may be congenital infection, particularly in preterm infants. This is associated with the lack of routine screening of the maternal genital tract, except for testing for group B streptococci during earlier stages of an uncomplicated pregnancy. **Case presentation:** This paper presents a case of a neonate with early-onset sepsis (EONS) caused by *Escherichia coli*. **Methods:** We carried out analysis of documents combined with the case report method. **Results:** Preterm birth associated with PPROM constitutes a significant risk factor for early-onset neonatal sepsis (EONS). **Conclusions:** The case illustrates the dynamic course of congenital infection and the resulting need for immediate, life-saving interventions. It also demonstrates abnormalities identified in ancillary investigations involving both the visual system and the central nervous system (CNS), confirming the intrauterine onset of the infectious process.

## 1. Introduction

Neonatal sepsis is defined as a clinical condition in which the presenting signs and symptoms, together with laboratory findings, are consistent with a systemic inflammatory response. It occurs as a result of infection caused by a variety of pathogens, including bacteria, viruses, and fungi [1]. This condition leads to multiorgan dysfunction with organ failure and, consequently, may result in death within the first 28 days of life [2,3]. Taking into account the dynamics of infection development, early-onset neonatal sepsis (EONS), occurring within 72 h after birth, and late-onset neonatal sepsis (LONS), developing thereafter, are distinguished [4,5]. This classification is important for determining differences in etiology, diagnostic approach, and treatment outcomes in neonatal sepsis.

The most common pathogens responsible for neonatal sepsis are *Escherichia coli* (*E. coli*) and group B streptococci (GBS) [6,7]. *E. coli* is the predominant pathogen in sepsis among preterm infants, whereas GBS is more common in term neonates. *E. coli* is a Gram-negative, flagellar bacterium belonging to the family Enterobacteriaceae [8]. An important factor in the defense against Gram-negative bacteria is the activation of the Toll-like receptor 4 (TLR4) signaling pathway [9,10]. Upon contact of macrophages with Gram-negative bacteria, the lipid component of lipopolysaccharides (LPS) present in their outer membrane binds to MD2, resulting in activation of the TLR4 pathway through dimerization of TLR4 with MD2 [9]. Following TLR4 activation, there is upregulated expression of MyD88 (myeloid differentiation primary response 88), TIRAP (TIR domain-containing adaptor protein), TRAM (TRIF-related adaptor molecule), and TRIF (TIR domain-containing adaptor inducing interferon-β) [11,12]. This facilitates the recruitment and activation of TAK1, as well as phosphorylation of the IκB complex, promoting its degradation and the release of NF-κB [13]. TAK1 activates various mitogen-activated protein kinases (MAPKs) [14] and, together with NF-κB, induces the production and release of proinflammatory cytokines IL-1β, TNF-α, and IL-6 [15]. TLR2 and TLR4 play a particularly important role as key pattern recognition receptors for pathogen-associated molecular patterns (PAMPs) in the neonatal immune system. In preterm infants, reduced TLR expression and impaired signal transduction have been observed, resulting in decreased NF-κB activation and lower levels of proinflammatory cytokines, including IL-6 and TNF-α. Delayed IL-6 production following exposure to GBS has also been reported, indicating functional immaturity of the neonatal innate immune response [16,17]. The most severe among PAMP/TLR4-mediated diseases is sepsis [18]. Septic shock, characterized by elevated circulating LPS levels, excessive expression of proinflammatory cytokines, activation of the coagulation system, and accumulation of fibrin degradation products, leads to both local and systemic hemodynamic disturbances as well as endothelial dysfunction through the Toll-like receptor signaling pathway [19]. The cytokine cascade, involving the release of vasoactive and cytotoxic mediators, causes rapid multi-organ damage (MOF) [20]. Neonatal sepsis due to *E. coli* is frequently complicated by MOF and carries a high risk of mortality [21].

Therefore, through the presented case, we aimed to draw attention to the fact that the prevention of *E. coli*–associated sepsis represents a major clinical challenge [22]. Of particular concern are infections caused by ESBL-producing strains, which are associated with a twofold higher mortality compared with sepsis caused by ESBL-negative *E. coli*. These strains demonstrate significant antimicrobial resistance and may be associated with a rapidly progressive, fulminant course of the disease [23].

The aim of this report is to present a clinically significant case of EONS caused by ESBL-producing *E. coli* in a neonate born at our center. Our objective was to demonstrate the possibility of a rapidly progressive intrauterine infection in the neonate despite appropriately administered antibiotic prophylaxis in the setting of preterm premature rupture of membranes (PPROM) in the mother.

## 2. Case Presentation

A male neonate was delivered by caesarean section due to threatened intrauterine fetal asphyxia. He was born from the second pregnancy and second delivery at 32 + 1/7 weeks of gestation, with a birth weight of 1950 g (60th percentile), body length of 44 cm (74th percentile), and head circumference of 31 cm (82nd percentile). The infant was in good general condition at birth and received Apgar scores of 9 and 8 at 1 and 5 min, respectively.

Maternal history revealed a pregnancy complicated by preterm premature rupture of membranes with leakage of amniotic fluid lasting approximately 53 h. Cardiotocography (CTG) remained normal until qualification for caesarean section, after which a narrowed tracing with late decelerations was observed, prompting the decision to proceed with surgery due to threatened fetal asphyxia. The mother received magnesium sulfate for neuroprotection, prophylactic antibiotic therapy initiated two days before delivery (azithromycin and clindamycin in accordance with the recommendations of the Polish Society of Gynecologists and Obstetricians based on guidelines from the National Institute of Medicines, with metronidazole administered on the first day of treatment), and a full course of betamethasone [24,25]. After birth, noninvasive respiratory support with nasal continuous positive airway pressure (nCPAP) was initiated due to respiratory insufficiency. On physical examination, the neonate’s morphological and neurological findings were appropriate for the gestational age. Umbilical cord blood gas analysis was within normal limits. Given the obstetric history and gestational age, empiric combination antibiotic therapy with ampicillin and gentamicin was initiated, along with caffeine citrate.

During further observation in the NICU, due to worsening signs of respiratory failure requiring an increasing fraction of inspired oxygen (FiO_2_) up to 30% in the breathing mixture, the mode of respiratory support was changed to synchronized intermittent positive pressure ventilation—bilevel positive airway pressure (SIPAP–BiPhasic). At 3 h of life, because of persistent respiratory insufficiency with a continued need to increase FiO_2_ above 30%, surfactant was administered using the INSURE technique to ensure controlled delivery, followed by continuation of SIPAP–BiPhasic ventilation. After transient improvement, the patient’s general condition deteriorated again; therefore, at the beginning of the 6th hour of life, sedation with fentanyl (1.5 µg/kg/h) was introduced, the infant was intubated, and mechanical ventilation was commenced using pressure-controlled ventilation (PCV) with assist/control (AC) mode and volume guarantee (VG). Monitored vital parameters, apart from oxygen saturation, remained within normal ranges. As persistent pulmonary hypertension of the newborn (PPHN) could not be excluded, arrangements were made for transfer to a tertiary referral center for potential inhaled nitric oxide (iNO) therapy. Prior to transfer, treatment included a fluid bolus followed by hydrocortisone 8 mg and dobutamine at 10 µg/kg/min. A gradual deterioration in the patient’s condition was observed, and due to clinical worsening, the infant was transferred to the Neonatal Intensive Care Unit at Bielański Hospital.

Upon admission to the tertiary center at 8 h of life, the infant was in severe general condition. Treatment was escalated to include adrenaline (0.6–0.3–0.4 µg/kg/min), followed by noradrenaline (0.2–0.3 µg/kg/min), and iNO therapy at 20 ppm. Arterial blood gas analysis revealed hypoxemia and persistent metabolic acidosis. Consequently, milrinone at 0.5 µg/kg/min and sodium bicarbonate were added to therapy. Simultaneously, the infant developed hyperthermia up to 39 °C; paracetamol was administered with a moderate effect. At 15 h of life, the microbiology laboratory reported positive growth in the blood culture obtained at birth. At 32 h of life, Gram-negative rods were identified, and at 48 h, *Escherichia coli* ESBL-positive was confirmed (Table 1.).

Taking into account the presented findings, including the antimicrobial susceptibility profile based on the blood culture results, and given that meningitis could not be excluded, antimicrobial therapy was intensified. Meropenem was added at doses appropriate for central nervous system (CNS) infection, and gentamicin was discontinued.

During further hospitalization, ventilatory settings and pharmacotherapy were adjusted as needed. From day 4 of life, in view of progressive clinical improvement, adrenaline was discontinued, and after stabilization of the general condition, a lumbar puncture was performed for diagnostic purposes (direct smear negative; testing at the National Reference Centre for the Diagnosis of Bacterial Infections of the Central Nervous System was negative). On day 5 of life, iNO therapy was discontinued, and antifungal prophylaxis with fluconazole was initiated. Echocardiographic examination revealed a patent foramen ovale (PFO) with bidirectional shunting, a patent ductus arteriosus (PDA), and signs of moderately elevated pulmonary vascular resistance. On day 6 of life, the infant was extubated to non-invasive ventilation (NIV), and milrinone was discontinued. From day 7 of life, ventilation was switched to CPAP; from day 9 to high-flow nasal cannula (HFNC); and from day 11 of life, the neonate breathed spontaneously with intermittent passive oxygen therapy. A follow-up blood culture obtained on day 9 of life showed no bacterial growth. Antibiotic therapy was completed after 14 days. Follow-up echocardiographic examinations on days 8 and 18 of life demonstrated a patent PFO with left-to-right shunt and persistent features of moderately elevated pulmonary vascular resistance. On day 47 of life, a patent PFO with left-to-right shunt was still observed.

Additional complications observed during treatment included:

1. Feeding difficulties: Due to the patient’s critical condition, enteral feeding was withheld until day 6 of life. Enteral nutrition was initiated on day 7, with gradual advancement of feeding volumes. Until day 14 of life, parenteral nutrition via a central venous catheter was additionally administered; catheter position was regularly monitored using radiological imaging. Ultrasonographic examination of the abdominal organs remained normal and appropriate for age.
2. Hematological issues: Leukopenia was noted in the complete blood count obtained after birth. Due to anemia in the context of severe general condition, red blood cell transfusions were performed on days 3, 5, and 46 of life.
3. Neurological issues: Cranial ultrasound performed on day 4 of life revealed extensive hypoxic–ischemic malacic changes in both cerebral hemispheres, located in the parieto-occipital periventricular regions. On day 11 of life, based on ultrasound findings and the evolution of the lesions, it was suggested that the hypoxic–ischemic changes observed both in the current and previous examinations most likely originated during fetal life. To further extend the diagnostic evaluation, brain magnetic resonance imaging (MRI) was performed on day 34 of life. The examination revealed extensive intracerebral hemorrhagic lesions located in the left parieto-occipital region, right parieto-occital region, and the right temporal region, as well as above the cerebellar vermis. Numerous cavities lesions, likely of hypoxic–ischemic origin, were also observed adjacent to the medial and anterior portions of the lateral ventricles. A neurological consultation was conducted on day 35 of life; no abnormalities were identified. Muscle tone, flexor posture, and spontaneous movements were assessed as appropriate for age.
4. Ophthalmological issues: Fundoscopic examination suggested a possible infection occurring during the last period of pregnancy.

The infant was discharged home in good general condition on day 49 of life.

## 3. Discussion

In our article, we presented a case of EONS of ESBL-producing *E. coli* etiology, characterized by an exceptionally rapid course with numerous functional and anatomical complications in both the fetus and the neonate. Of particular interest is the simultaneous occurrence of clinical symptoms, namely the rise in body temperature at 16 h after delivery, as well as elevated inflammatory markers in both the mother and the infant (Table 2).

The above findings may support the diagnosis of both intrauterine infection and vertical transmission. Together with blood culture results and the clinical presentation, they form a comprehensive diagnostic framework and provided the basis for the therapeutic approach implemented.

Knowles et al. and Bhattacharjee et al. identified *E. coli* as a leading cause of neonatal meningitis and sepsis, posing a significant risk to both maternal and fetal health [28,29]. Simultaneously, Shi et al. emphasize that effective screening tools and preventive measures are currently lacking, and studies investigating the impact of these infections on mothers and fetuses remain limited [30]. In line with this evidence, our article addresses the ongoing issue of intrauterine infection risk in women with threatened preterm labor and PPROM. Similarly, Schrag et al. identified prematurity, low birth weight, and maternal chorioamnionitis as specific risk factors for *E. coli*-associated EONS [31]. Confirmation of these findings is also reported by Tsai et al., who, as in our case, observed that the occurrence of sepsis symptoms within the first 24 h of life in neonates born to preterm mothers with PPROM and intrapartum fever is particularly characteristic of *E. coli* etiology, significantly more so than for other pathogens [32]. A similar conclusion was drawn by Lai et al. [33]. The failure of antibiotic prophylaxis in these conditions among pregnant women can be attributed to the antimicrobial resistance of many pathogens. This observation aligns with the study by Villavicencio-Carrisoza et al., which highlights the compounding effect of virulence factors and antibiotic resistance genes in ESBL and XDR strains, further emphasizing the challenges of managing infections with limited antibiotic options and the importance of stringent infection control practices [7]. In the presented case, a combined antibiotic regimen was administered to the neonate for a duration of 14 days. This management approach is consistent with the guidelines of the American Academy of Pediatrics [34]. At the same time, the issue of appropriate prophylactic management in women with PPROM between 24 and 33 weeks of gestation deserves attention. According to the most recent ACOG guidelines, management may involve either expectant observation or prompt delivery, with careful and continuous monitoring in a hospital setting. In addition, the importance of antenatal steroid therapy, fetal neuroprotection with magnesium sulfate, and prophylactic antibiotic therapy during the latency period is emphasized. Nevertheless, as stated in the guidelines, there is no evidence supporting a single optimal antibiotic regimen during latency [35]. These recommendations reflect the challenges encountered in our case analysis, which clearly demonstrated that, despite adherence to the most current and widely accepted guidelines, it is not possible to fully protect either the fetus or the neonate from infection. Preterm neonates, in particular, exhibit a reduced capacity to mount effective responses to bacterial ligands such as lipopolysaccharide (LPS) and lipoteichoic acid. This impaired recognition can lead to delayed pathogen clearance, followed by the development of exaggerated systemic inflammation. These observations are consistent with previous literature documenting the increased susceptibility to sepsis among preterm populations [3,36].

The neonatal immune system is further compromised by its immaturity, reduced neutrophil activity, lower complement levels, and a predisposition toward immunological tolerance [37,38]. At the same time, the role of sterile inflammation in the pathogenesis of EONS is emphasized, involving immune activation without direct infection [39,40,41]. Factors such as mechanical ventilation, prenatal inflammatory states, and hypoxia–reperfusion injury can trigger the release of damage-associated molecular patterns (DAMPs), including HMGB1 (high-mobility group box 1 protein), adenosine triphosphate (ATP), and mitochondrial DNA [42,43,44].

Key elements contributing to multi-organ dysfunction in EONS include endothelial injury, coagulation disturbances, and mitochondrial dysfunction [45,46,47]. The complex pathophysiology blurs the distinction between infectious and non-infectious causes, further complicating clinical decision-making [48].

Advances in molecular diagnostic techniques, such as microRNA analysis, transcriptomic profiling, proteomics, and immune checkpoint profiling, have revealed novel biomarkers and potential therapeutic targets for the early detection of EONS [49,50]. Recent studies have identified distinct gene signatures associated with sepsis, dysregulated microRNAs, and novel cell surface markers, which may enhance risk stratification and facilitate the development of individualized treatment strategies.

The mode of pathogen transmission is also critical. Hwang et al. highlighted that transmission primarily originates from the maternal vaginal microbiota, with *E. coli* being the most frequently transmitted pathogen to neonates. Maternal leukocytosis, histologically confirmed chorioamnionitis, and a history of cervical inflammation were identified as contributing factors. In very-low-birth-weight infants (VLBWI), decreased buffering capacity and elevated serum CRP levels were associated with pathogen transmission. The study also noted that vertical transmission correlated with increased mortality and a higher incidence of grade III or higher intraventricular hemorrhage in neonates [51].

Furthermore, Stoll et al. and Glaser et al. raised the issue that despite advances in neonatal care practices—resulting in improved survival rates among preterm infants and increased maternal antibiotic use in recent years—there has been no significant change in the incidence of EONS [52,53]. This is likely due to the susceptibility of VLBWI to infections caused by colonizing bacteria, reflecting their underdeveloped immune systems. Considering that their microbiota is largely derived from the mother before and during delivery, the transmission of aberrantly colonizing maternal pathogens poses an increased risk of EONS [51]. Therefore, developing effective methods to assess the risk of vertical pathogen transmission in these infants is of critical importance.

The presented case is particularly significant because it demonstrates not only the simultaneous, correlated occurrence of clinical signs in both the mother and the neonate, but also highlights the possibility of EONS caused by ESBL-producing *E. coli* in a neonate whose mother, apart from PPROM, showed no additional signs of infection prior to delivery. Consequently, in this case, despite adherence to the ACOG guidelines for the management of PPROM at 32 weeks of gestation, we observed intrauterine infection in the fetus leading to EONS [35]. This underscores the importance of a holistic approach to diagnosis and treatment, with careful consideration of epidemiological and statistical data. At the same time, it emphasizes that prophylaxis does not equate to complete prevention of infection. Therefore, even when clinical management follows both current ACOG guidelines and established standards of neonatal care, the possibility of infection must always be anticipated.

## 4. Conclusions

This case highlights the extreme life-threatening risk for a neonate with congenital infection, likely accompanied by subsequent anatomical alterations, including pathological findings in the CNS and ocular structures, despite a normal CTG tracing up to the point of qualification for cesarean delivery. A major challenge remains the development of an evidence-based protocol for screening, prophylaxis, and treatment, including the selection of appropriate antimicrobial agents, dosing, and routes of administration for women at risk of preterm delivery in the setting of PPROM, with the aim of effectively preventing intrauterine infection.

## Figures and Tables

**Table 1:** Assessment of the strain’s resistance profile (ESBL/MDR).

Antibiotic/Antifungal	Susceptibility	MIC ^a^/BP ^b^
Amoxicillin/Clavulanic Acid	S ^c^	4
Piperacillin/Tazobactam	S	≤4
Cefuroxime iv	R ^d^	>32
Ceftriaxone	R	>32
Cefotaxime (Meningitis)	R	>32
Cefotaxime (Other)	R	>32
Ceftazidime	R	8
Cefepime	R	16
Imipenem	S	≤0.25
Meropenem (Meningitis)	S	≤0.25
Meropenem (Other)	S	≤0.25
Amikacin	S	≤2
Gentamicin	S	≤1
Tobramycin	S	≤1
Ciprofloxacin	R	>2
Tigecycline	S	≤0.5

^a^ MIC—Minimum Inhibitory Concentration. ^b^ BP—Breakpoint. ^c^ S—Susceptible. ^d^ R—Resistant.

**Table 2:** Summary of inflammatory markers in the mother and the neonate at successive hours of the infant’s life.

	Neonatal Laboratory Results						Maternal Laboratory Results								Reference Range [26,27]	Unit
Time	8 *	12	50	120	168	198	288	−3	24	38	72	96	168	216	-	hour
CRP	0.6	1.5	10	1.4	0.5	0.3	0.1	0.5	14	22	16	10	5	2	0.01–0.64 at birth 0.3–11.0 at 27–36 h 0.1–4.7 at 90 h 0.7–32.0 at 120 h	mg/L
Procalcitonin	-	-	-	50	5.5	1.7	0.4	-	-	0.6	0.4	-	-	-	0.01–0.56 at birth 0.9–48.4 at 21–22 h 0.01–0.8 at 120 h	ng/mL
Leukocytes	-	1.2	11	17	27	21	16	12	12	12	9	7.5	11	8	2.3–7.4 at birth 2.6–7.1 at 24 h 1.5–6.2 at 48 h 2–6.2 at 72 h 2.2–6.6 at 96 h	tys/ul

* Hour “0”—time of delivery.

## Data Availability

All data supporting the results are included within the article, and no additional or source data are required.
